# Non-Coding RNAs in Psychiatric Disorders and Suicidal Behavior

**DOI:** 10.3389/fpsyt.2020.543893

**Published:** 2020-09-15

**Authors:** Yuta Yoshino, Yogesh Dwivedi

**Affiliations:** Department of Psychiatry and Behavioral Neurobiology, University of Alabama at Birmingham, Birmingham, AL, United States

**Keywords:** major depressive disorder, schizophrenia, bipolar disorder, long non-coding RNAs, microRNAs

## Abstract

It is well known that only a small proportion of the human genome code for proteins; the rest belong to the family of RNAs that do not code for protein and are known as non-coding RNAs (ncRNAs). ncRNAs are further divided into two subclasses based on size: 1) long non-coding RNAs (lncRNAs; >200 nucleotides) and 2) small RNAs (<200 nucleotides). Small RNAs contain various family members that include microRNAs (miRNAs), small interfering RNAs (siRNAs), piwi-interacting RNAs (piRNAs), small nucleolar RNAs (snoRNAs), and small nuclear RNAs (snRNAs). The roles of ncRNAs, especially lncRNAs and miRNAs, are well documented in brain development, homeostasis, stress responses, and neural plasticity. It has also been reported that ncRNAs can influence the development of psychiatric disorders including schizophrenia, major depressive disorder, and bipolar disorder. More recently, their roles are being investigated in suicidal behavior. In this article, we have comprehensively reviewed the findings of lncRNA and miRNA expression changes and their functions in various psychiatric disorders including suicidal behavior. We primarily focused on studies that have been done in *postmortem* human brain. In addition, we have briefly reviewed the role of other small RNAs (*e.g.* piwiRNA, siRNA, snRNA, and snoRNAs) and their expression changes in psychiatric illnesses.

## Introduction

Non-coding RNAs (ncRNAs) are defined as RNAs that are not translated into protein. Protein-coding genes occupy only a small proportion (<3%) of the entire genome (1). However, the remaining non-protein coding genes are not a simple transcriptional noise (1, 2); instead, ~80% of them are transcriptionally active with elaborate regulatory roles. A majority of them are now considered non-coding RNA genes (3–6). Based on the size of the nucleotides, ncRNAs are divided into small ncRNAs and long ncRNAs (lncRNAs). ncRNAs that contain <200 nucleotides (nt) belong to small ncRNA family. On the other hand, those containing more >200 nt belong to long lncRNA family. Small RNAs include microRNAs (miRNAs), small interfering RNAs (siRNAs), piwi-interacting RNAs (piRNAs), small nucleolar RNAs (snoRNAs), and small nuclear RNAs (snRNAs). ncRNAs function *via* interactions with RNA, DNA, and protein. They regulate the transcription of mRNAs and participate in alternative splicing and epigenetic modifications such as chromatin and RNA editing (7, 8). These regulatory functions can target either neighboring transcripts (cis) or loci that are distant from their own transcription (trans). Collectively, ncRNAs constitute a unique layer of gene regulation where they function as key intermediate regulators in conveying the message from genotype to phenotype states (9).

A large number of ncRNAs are abundantly expressed in the brain (10–12). In addition, the expressions of ncRNAs are brain region- and cell type-specific (13–16). Several studies have shown the role of ncRNAs in brain evolution, development, homeostasis, stress response, and neuroplasticity (14, 17–25). Brain expressed ncRNAs can also influence the development of psychiatric disorders such as schizophrenia (SCZ), major depressive disorder (MDD), and bipolar disorder (BD) as well as neurodegenerative disorders (11, 19, 23, 26, 27). More recently, their role in suicidal behavior has been postulated (28–30). The effects of ncRNA expression changes are not restricted to the type of psychiatric disorders but, to a large extent, to different brain regions. Recent evidence suggests the inability of brain regions to act independently. Instead, they act in a coordinated manner *via* functional networks. In various neuropsychiatric disorders, voxel based neuroimaging has demonstrated specific roles of brain regions such as cortex (prefrontal, anterior cingulate), hippocampus, and amygdala (31). Although each of these brain regions has important roles, such as amygdala and prefrontal cortex in emotion (32) and prefrontal cortex and hippocampus in stress response (33), these brain regions crosstalk to each other *via* complex gene networks which could be mediated *vis* ncRNAs.

Earlier published reviews have primarily focused on miRNAs in psychiatric illnesses (34–36). This review comprehensively summarizes the relationship between various ncRNAs. Although the emphasis is given to lncRNAs and miRNAs, a range of other ncRNAs are also included to provide a glimpse into where the field stands and what should be the course of direction for future research. The review is restricted to studies in human *postmortem* brain, given that the role of peripheral ncRNAs is not very well established. We have discussed a few peripheral blood cell studies which are relevant to suicidal behavior. The criteria for selecting literature search are as follows: Electronic search was done using PubMed with search terms ncRNAs (microRNA, small interfering RNA, piwi-interacting RNA, small nucleolar RNA, and small nuclear RNA) AND psychiatric disorders (schizophrenia, major depressive disorder, and bipolar disorder). Each combination was performed separately. Additionally, *postmortem* research for non-suicide studies, and both *postmortem* brain and peripheral tissue research for suicide studies, were included in the search criteria.

## Long Non-Coding RNAs

### Long Non-Coding RNAs in Brain Functions

**Figure 1:**
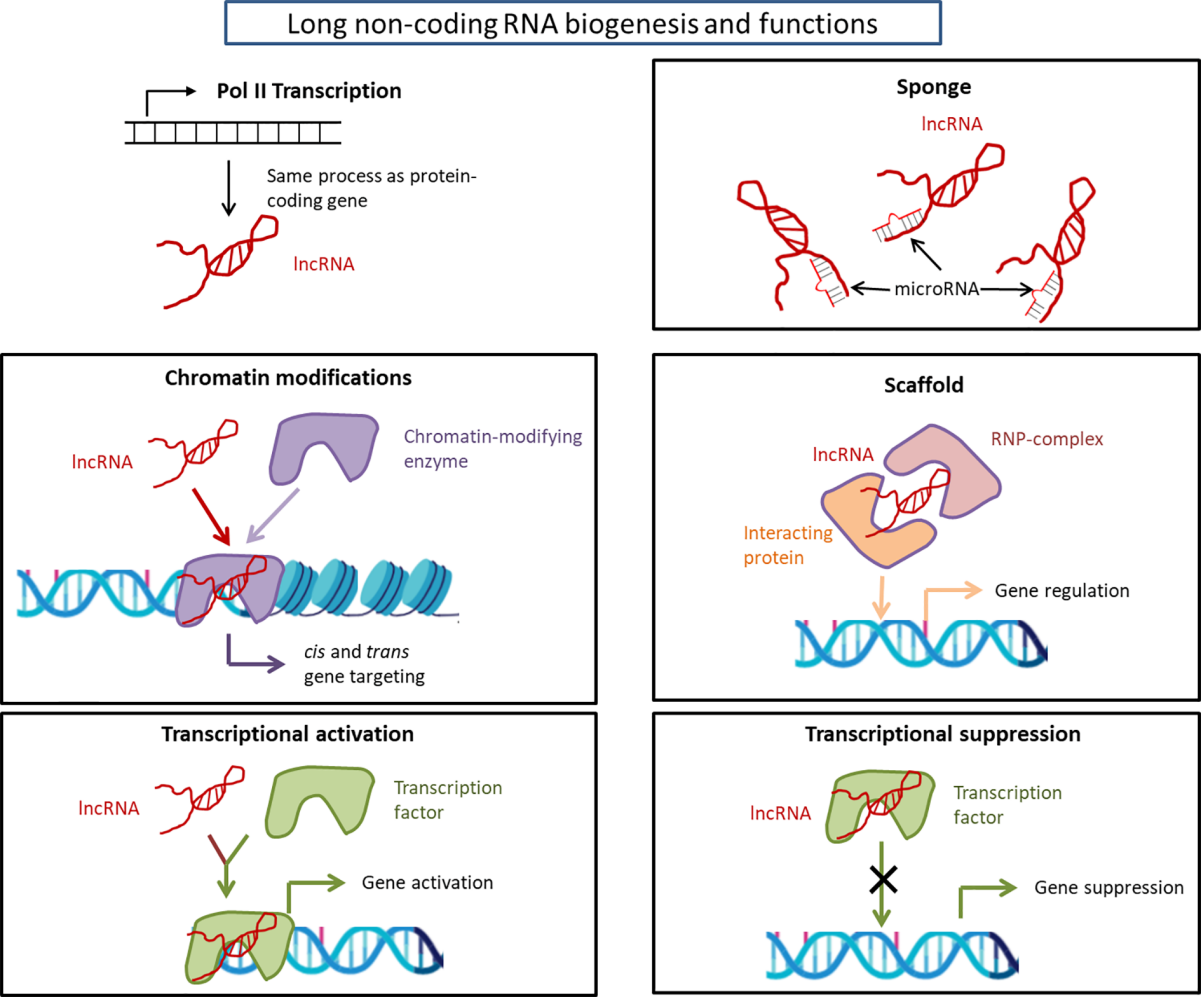
Long non-coding RNA biogenesis and functions. Long non-coding RNAs are transcribed by RNA polymerase II and are processed to mature RNA by the same method as protein-coding mRNAs as detailed in the text. The considerable functions of lncRNAs are: chromatin modification, transcriptional activation/suppression, sponge, and scaffold. The functions of lncRNAs depend on the type of lncRNAs. lncRNA, long non-coding RNA; RNP, ribonucleoprotein.

Long non-coding RNAs (lncRNAs) are defined as RNAs having >200 nucleotides with low (in the form of short peptide) or no protein coding potential (37). LncRNAs are transcribed by RNA Polymerase II and are processed to mature RNA by the same method as protein-coding mRNAs (38). LncRNAs’ lengths span ~100 kilo base-pairs (bp) and are shorter than mRNAs but have longer exons (39–41). They were originally considered to be the products of heterochronic genes (4) that control the temporal dimension of development; however, there are limitations to fully understand the function of lncRNAs from this point of view. Interestingly, the genomic structure of lncRNAs is strongly related to their function (42). In the early nineties, the X-inactive specific transcripts (Xist) and H19 were first discovered as non-protein coding RNAs by exploring cDNA libraries (43, 44). Since then, over 100,000 lncRNA genes have been found and their number is still increasing (45). Recently, several lncRNA functions have been revealed. For example, they participate in chromatin modifications (46, 47); act as ‘sponges’ that prevent miRNA functions (48); act as scaffolds that provide docking sites for proteins (49); serve as activators and suppressors of mRNA transcription (50); and act as regulators of splicing patterns (50). The functions of lncRNAs are depicted in **Figure 1**.

In terms of functions in the brain, lncRNAs are strongly related to brain development (51). In this capacity, lncRNAs participate in spatiotemporal regulation of proliferation and differentiation of pluripotent stem cells (21, 52). High-resolution and high-throughput technologies, such as microarray and RNA-sequencing, have helped in examining the number and pattern of lncRNA expression in the brain. Microarray detects the expression of a large number of RNAs simultaneously, whereas RNA-seq detects both known and novel transcripts and quantifies a large dynamic range of expression levels with absolute rather than relative values. Ramos et al. (53) reported that more than 3,600 lncRNAs are expressed among three different brain regions: subventricular zone, olfactory bulb, and dentate gyrus. Clustering analysis revealed that lncRNAs have a tissue-specific expression pattern (39, 54). A study, using microarray analysis, investigated differential lncRNA expression in the temporal cortex ranging from infancy to adulthood (0.92–43.5 years old) and concluded that the expression pattern of lncRNA changes with age (55). At functional levels, several lncRNAs are involved in regulating neuronal development. This occurs through complex interactions between lncRNAs and other factors in transitioning neural stem cell to progenitors and eventually to differentiated neurons. Some of the lncRNAs that promote the differentiation of neural stem cells include Brn1b, RMST, and TUNA (56–58). EVF2, the first found central nervous system-specific lncRNA, regulates the differentiation of GABAergic neurons (18). The GABA system has been shown to be consistently involved in several psychiatric disorders such as SCZ (59–61), MDD (62), and BD (63). Another lncRNA, Pnky, promotes neural proliferation (64, 65). A recent study has shown that antisense lncRNAs also regulate key proteins related to psychiatric disorders. An inhibition of brain-derived neurotrophic factor (BDNF) antisense RNA (BDNF-AS) causes two- to sevenfold increase in BDNF protein level, which leads to neuronal outgrowth, differentiation, survival, and proliferation (66). An interaction between BDNF and neuropsychiatric disorders is well documented (67, 68). In addition, the mechanism of action of antidepressants (69) and electroconvulsive therapy (70) also involve changes in the levels of BDNF, both centrally and peripherally. Not only do lncRNAs impact BDNF, but changes in the expression of a large number of lncRNAs have been shown following 1 h BDNF treatment in cultured neuronal cells (71). This shows that under neuronal environment, a feed forward and feedback loop might exist between neurotrophin signaling and lncRNA expression with the potential to develop effective therapeutic strategy in psychiatric conditions.

### Long Non-Coding RNAs in Psychiatric Disorders

Examining the role of ncRNAs in psychiatric illnesses is an emerging field. Two recent studies used amygdala from SCZ subjects to decipher the role of lncRNAs in this disorder (**Table 1**). Tian et al. (72) examined lncRNA expression by RNA-seq using 21 SCZ and 24 healthy control subjects. They found alterations in the expression of 345 lncRNAs (104 up- and six downregulated) in SCZ subjects. Subsequently, they divided 21 SCZ subjects into nine undifferentiated, seven disorganized, and five paranoid. Individually, they found 155 up- and 17 downregulated lncRNAs in the undifferentiated SCZ group, 39 up- and six downregulated lncRNAs in the disorganized SCZ subjects, and no significant alteration in paranoid SCZ subjects when compared with control subjects. They performed gene set enrichment analysis to reveal the gene ontology terms associated with dysregulated lncRNAs (all SCZ *vs* control subjects) and found that dysregulated lncRNAs were associated with protein synthesis, blood vessel development, nervous system pathways, and immune system pathways. In another amygdala study, Liu et al. (73) used RNA-seq for detecting dysregulation of lncRNAs in 22 SCZ subjects and 24 healthy controls. Transcriptome data identified 250 lncRNAs that showed significant expression differences between SCZ and control subjects. The study further focused on two specific lncRNAs (upregulated RP11-724N1.1 and downregulated RP11-677M14.2), which were shown to be associated with SCZ based on previous genome-wide association study (GWAS). Additional analysis by dividing SCZ subjects into three subtypes revealed the dysregulation of RP11-724N1.1 in the undifferentiated subtype and RP11-677M14.2 in the paranoid subtype. RP11-724N1.1 overlaps the CNNM2 gene locus, which has a key role in the neurodevelopmental process (74). On the other hand, RP11-677M14.2 overlaps the neurogranin (NRGN) gene locus. Several SNPs of the NRGN gene have been associated with onset (75–77), intellectual disability (75), and anterior cingulate cortex (ACC) volume (78) in SCZ subjects. These amygdala studies suggest that not only are lncRNAs involved in SCZ pathophysiology, but they can also differentiate subtypes of SCZ.

**Table 1:** LncRNA expression changes in psychiatric disorders based on *postmortem* brain studies.

Brain areas	Sample size	Methods	lncRNA expression changes		lncRNA functions and roles in disease conditions	Citations
			Upregulation	Downregulation		
Amygdala	21 SCZ and 24 Controls	RNA-seq	104 lncRNAs	AC140542.2, AP001347.6, LINC01011, SOCS2-AS1, AP003774.4, AC004540.5	Dysregulated lncRNAs were associated with protein synthesis, blood vessel development, nervous system pathways, and immune system pathways	(72)
Amygdala	22 SCZ and 24 Controls	RNA-seq	170 lncRNAs	80 lncRNAs	Two important lncRNAs (RP11-724N1.1 and RP11-677M14.2) located in the regions previously associated with SCZ based on GWAS are included	(73)

lncRNA, long non-coding RNA; SCZ, Schizophrenia.

### lncRNAs and Suicidal Behavior

There are four studies that show a relationship between suicidal behavior and lncRNA expression changes. Of them, three studies explored lncRNA expression in the brain (one in ACC and two in the dorsolateral prefrontal cortex [dlPFC]), and one in the blood (**Table 2**). Zhou et al. (79) compared lncRNA expression levels in rostral ACC between 26 MDD suicide and 24 healthy control subjects by RNA-seq. After considering nominal *p* values (*p* < 0.05), without multiple testing, 364 out of 2,670 lncRNAs were differentially regulated; of them, 60% (217 lncRNAs) were downregulated. After genome-wide multiple testing, 15 upregulated and eight downregulated lncRNAs reached statistical significance. Potential cis-targets for significantly dysregulated 23 lncRNAs based on RNA-seq data showed that six lncRNAs (RP11-326I11.3 *vs* IRF2; RP11-273G15.2 *vs* LY6E; CTD-2647L4.4 *vs* HMOBX1; CTC-487M23.5 *vs* REEP5; RP1-269M15.3 *vs* PTPRT; RP11-96D1.10 *vs* NFATC3) were significantly correlated with one antisense or overlapping gene. All of them were found to be nominally differentially expressed except REEP5. With an additional qPCR approach, the authors validated three out of five genes (IRF2, LY6E, and HMBOX1). These genes are functionally related to interferon signaling, which plays a key role in CNS homeostasis and in psychiatric disorders (81).

**Table 2:** LncRNA expression changes in *postmortem* brain of suicide subjects.

Brain areas and blood source	Sample size	Methods	lncRNA expression changes		lncRNA functions and roles in disease conditions	Citations
			Upregulation	Downregulation		
ACC	26 MDD suicide and 24 Controls	RNA-seq and qPCR validation	SNORD3C, LLNLF-65H9.1, RP1-269M15.3, AC006003.3, AC012507.3, AC013460.1, C9orf106, DYX1C1-CCPG1, RP1-63G5.5, RP11-1186N24.5, RP11-1391J7.1, RP11-143K11.5, RP11-273G15.2, RP11-434C1.1 ZNF833P	RP11-453F18_B.1, RP11-96D1.10, AC004019.18, CTC-487M23.5, CTD-2647L4.4, RP11-326G21.1, RP11-326I11.3, RP11-326I11.5	The target genes Interferon regulatory factor 2 (IRF2, NM_002199), Lymphocyte antigen 6E (LY6E, NM_002346), and heme oxygenase (decycling) 1 (HMBOX1, NM_002133) (RP11-326I11.3, RP11-273G15.2, and CTD-2647L4.4) were successfully validated by qPCR	(79)
dlPFC	101 non-suicide, 50 non-violent suicide, 77 violent suicide (SCZ, MDD, and BD)	RNA-seq	LINC01268		Violent suicide group and aggressiveness group had higher expression of LINC01268	(28)
dlPFC	77 non-suicide, 13 non-violent suicide, and 16 violent suicide (SCZ)	RNA-seq			LOC285758 expression was associated with violent suicide	(80)
PBMC	63 MDD non-suicidal ideation, 57 MDD suicidal ideation, and 63 Controls	qPCR		TCONS_00019174, ENST00000566208, NONHSAG045500, ENST00000517573, NONHSAT034045, NONHSAT142707		(30)

ACC, Anterior Cingulate Cortex; BD, Bipolar Disorder; dlPFC, dorsolateral prefrontal cortex; lncRNA, long non-coding RNA; MDD, Major Depressive Disorder; PBMC, Peripheral Blood Mononuclear Cell; SCZ, Schizophrenia.

Punzi et al. (80) revealed a correlation between violent suicide and lncRNA LOC28758 expression in dlPFC of SCZ subjects that included 77 non-suicides, 13 non-violent suicides, and 16 violent suicides. LOC28758 is located in the MARCKS gene, which was also found to be significantly highly expressed in violent suicides than non-suicides and non-violent suicides. In a recent study, the authors also examined lncRNA expression changes in dlPFC by RNA-seq (28). LINC01268 expression was significantly higher in suicide subjects compared to non-suicide subjects. Note that both suicide and non-suicide groups included three psychiatric disorders (SCZ, MDD, and BD). Subsequently, they divided the suicide groups into violent suicide and non-violent suicide (poisoning or asphyxia = nonviolent; others = violent). The violent suicide group had higher expression of LINC01268 than the non-suicide group, but the non-violent group did not show any change. Additionally, they explored quantitative trait locus (eQTL) of LINC01268 by genotyping 23 SNPs located within 100 kb up and downstream of the gene coordinates. Of those, only rs7747961 could regulate the LINC01268 expression (C-carriers > non-C-carriers). Based on the Brown–Goodwin questionnaire score, they revealed that LINC01268 expression was higher in the aggressive group than in the non-aggressive group. Lastly, weighted correlation network analysis (WGCNA) was performed with RNA-seq data to verify the relationship between LINC01268 and the co-expression gene set. WGCNA is a systems biology method for detecting the modules correlated with clinical traits by a soft-threshold algorithm. A module including 224 genes and LINC01268 was created. It was found that among 224 genes, P2RY13, a purinergic receptor present in both the peripheral immune system and in the brain, had a strong correlation with LINC01268.

One peripheral blood mononuclear cell (PBMC) study was conducted to identify change in lncRNAs in MDD suicide subjects (63 MDD no-suicidal ideation, 57 MDD with suicidal ideation, and 63 control subjects) by qPCR method (30). qPCR method identifies amplified fragments during the PCR process, but only a smaller number of genes can be measured at once compared to RNA-seq or microarray. Six lncRNAs (TCONS_00019174, NST00000566208, NONHSAG045500, ENST00000517573, NONHSAT034045, and NONHSAT142707), previously confirmed to be downregulated in MDD subjects, were selected for this study. All six lncRNAs were downregulated in MDD with suicidal ideation, compared with MDD no suicidal ideation and control groups. The authors concluded that six lncRNAs may possibly serve as biomarker for suicidal ideation among MDD patients. Another study showed that decreased expression of TCONS_00019174 was associated with depression and expression change was rescued by antidepressant treatment (82). Additionally, an *in vitro* experiment showed that TCONS_00019174 was negatively correlated with phosphorylated-GSK3*β* (p-GSK3*β*) protein and *β*-catenin. Further, the overexpression of NONHSAG045500 inhibited the expression of serotonin transporter (SERT), while siRNA interference of NONHSAG045500 induced the upregulation of SERT (83). These studies indicate that the peripheral expression change of lncRNAs can have functional relevance and may be worth pursuing in the brain.

### microRNAs

**Figure 2:**
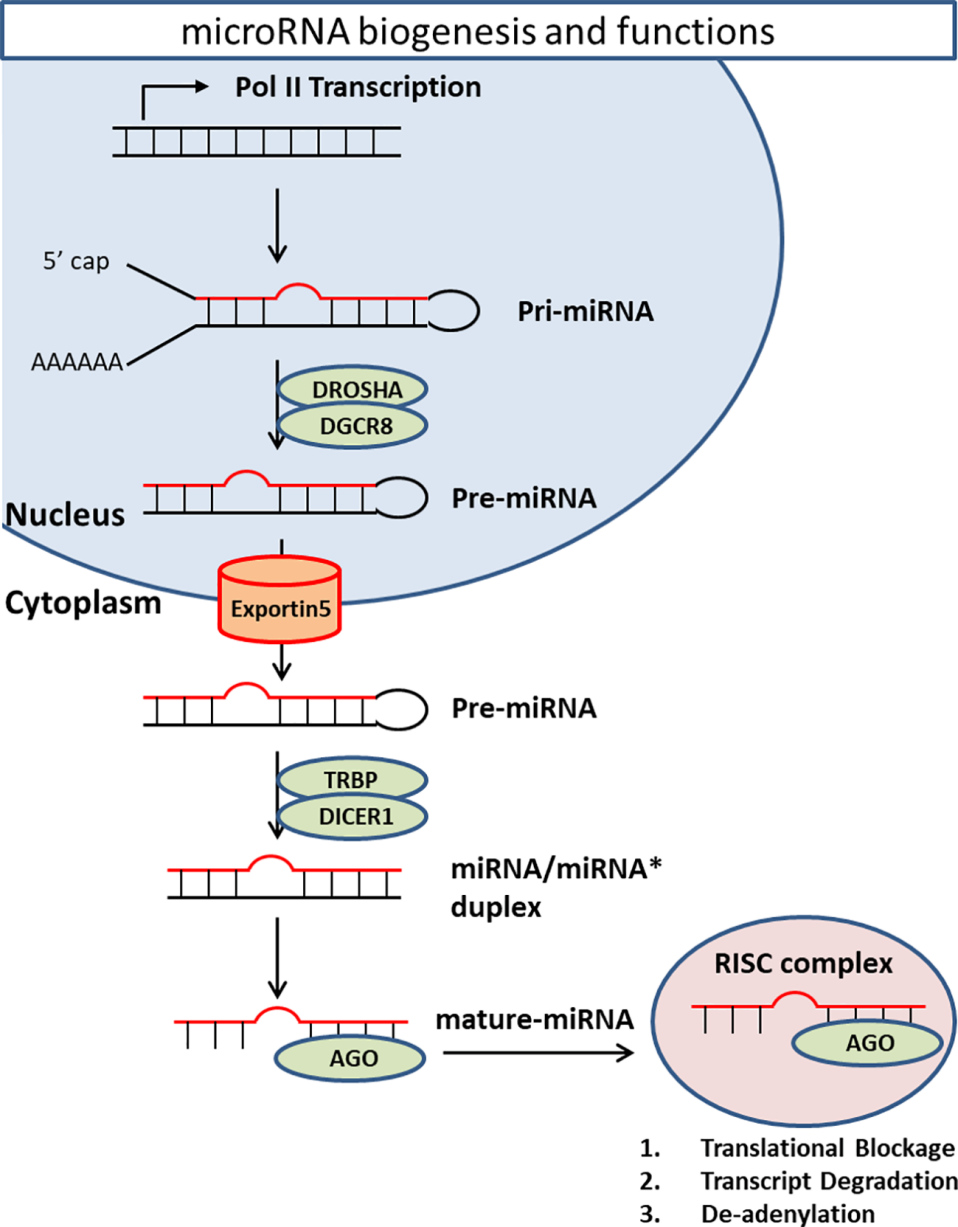
MiRNA biogenesis and functions. Primary miRNA is transcribed from encoded gene. RNase III enzyme Drosha generates precursor miRNA by removing the flanking segments and -11 bp stem region of pri-miRNA. Drosha requires DiGeorge syndrome critical region 8 (DGCR8) to complete this process. pre-miRNAs are then transported from nucleus to cytoplasm by a transporter Exportin-5 (XPO5) in a Ran-GTP-dependent manner. In the cytoplasm, Dicer converts pre-miRNA into double-strand mature miRNA. Dicer requires TAR RNA-binding protein (TRBP) as a cofactor. Finally, one strand of miRNA/miRNA* duplex loads onto Argonaute homolog protein (Ago) to make RNA-induced silencing complex (RISC). RISC/miRNA complex mainly works as suppressor of mRNA expression through translational blockage, transcript degradation, and deadenylation.

MicroRNAs (miRNAs) are the best studied ncRNAs in terms of functionality and relevance to psychiatric illnesses. Averaging 22 nucleotides in length, miRNAs are synthesized *via* several enzymatic processes. Initially, primary miRNA (pri-miRNA) is transcribed from an encoded gene. Next, RNase III enzyme Drosha generates precursor miRNA (pre-miRNA) by removing the flanking segments and -11 bp stem region of pri-miRNA. Drosha requires DiGeorge syndrome critical region 8 (DGCR8) to complete this process. pre-miRNAs are then translocated to cytoplasm with the help of Exportin-5 (XPO5). In the cytoplasm, dicer, a double-stranded RNA endoribonuclease, converts pre-miRNA into double-stranded mature miRNA. Dicer requires TAR RNA-binding protein (TRBP) as a cofactor (84–88). Finally, one strand of miRNA/miRNA* duplex loads into Argonaute homologue protein (Ago) to make RNA-induced silencing complex (RISC). RISC/miRNA complexes mainly work as suppressors of mRNA expression (**Figure 2**).

Several miRNAs play a key role in modulating synaptic functions and neural structures. Overexpression of miR-125b can disrupt spine structure, whereas sponging of endogenous miR-125b can induce the prolongation of dendritic protrusions (89). Introduction of miR-132 in hippocampal neurons can induce dendrite morphogenesis, which is caused by the regulation of GTPase-activating protein (p250GAP) (90, 91). At the behavioral level, transgenic miR-132 mice show altered cognitive functions through repression of MeCP2 translation, which is implicated in Rett Syndrom and mental retardation (92). The role of miRNAs in various psychiatric illnesses has been studied extensively. A summary of the human *postmortem* brain miRNA findings in psychiatric illnesses is provided in **Table 3**. Below, we discuss miRNA studies in human brain pertaining to SCZ, MDD, and BD as well as suicidal behavior.

**Table 3:** miRNA expression changes in psychiatric disorders based on *postmortem* brain studies.

Disease	Brain areas	Sample size	Methods	miRNA expression changes		Successfully validated miRNAs by qPCR	miRNA roles and other findings	Citations
				Up-regulation	Down-regulation			
**SCZ**	Amygdala	22 SCZ and 24 Controls	miRNA-seq	miR-196a-2, -1975, -34c, -451, -34a, -375, -144	miR-663, -639, -132, -124-2, -212, -483, -886, -585, -424, -520d, -1307		miR-1307 locus included rs11191419 previously reported in GWAS	(73)
	BA9	13 SCZ, 2 schizoaffective, and 21 Controls	Microarray	miR-106b	miR-26b, -30b, -29b, -195, -92, -30a-5p, -30d, -20b, -29c, -29a, -212, -7, -24, -30e, -9-3p	miR-26b, -30b, -92, -24, -30e		(93)
	BA9	35 SCZ, 33 BD, and 33 Controls	qPCR	19% of 234 miRNAs and 18 small nucleolar RNAs were changed due to SCZ or BD				(94)
	BA9	8 SCZ, 9 BD, and 13 Controls	Microarray	miR-497		miR-497	Exosome sample	(95)
	BA9	15 SCZ and 15 Controls	Microarray	Let-7d, miR-101, -105, -126*, -128a, -153, -16, -181a, -181d, -184, -199a, -20a, -219, -223, -27a, -29c, -302a*, -302b*, -31, -33, -338, -409-3p, -512-3p, -519b, -7		Let-7d, miR-128a, -16, -181b, -181a, -20a, -219, -27a, -29c, -7	Primary- and precursor-miR-181b and miRNA biogenesis-related enzymes were altered	(96)
	BA46	35 SCZ, 35 BD, and 35 Controls	TLDA	miR-34a, -132, -132*, -212, -544, -7, -154*				(97)
	BA46	37 SCZ/schizoaffective and 37 Controls	Microarray	miR-519c, -489-3p, -652, -382, -532, -199a*, -17-5p, -542-3p, -199b, -592, -495, 487a, -425-5p, -152, -148b, -134, -150, -105, -187, -154, -767-5p, -548b, -590, -502, -452*, -25, -328, -92b, -433, -222	miR-512-3p, -423, -193a	miR-17, -107, -134, -328, -382, -652	miRNA biogenesis-related enzymes were upregulated	(98)
	BA46	35 SCZ, 32 BD, and 34 Controls	qPCR		miR-346			(99)
	BA46	34 SCZ and 109 Controls	miRNA-seq	miR-3162, -936			The correlation-based hierarchical clustering analysis made nine miRNA groups	(100)
	dlPFC	35 SCZ, 31 BD, and 34 Controls	Microarray		miR-132, -132*		miR-132 was downregulated by NMDA antagonist in mice study	(101)
	dlPFC and several regions	1^st^ set (7 SCZ, 9 BD, and 10 Controls) 2^nd^ set (35 SCZ, 34 BD, and 35 Controls)	qPCR	miR-137 was not significantly changed				(102)
	STG	15 SCZ and 15 Controls	Microarray	59 of 274 (21%) miRNAs		let-7e, miR-107, -15a, -15b, -16, -195, -181b, -20a, -26b, -19a		(96)
	STG	21 SCZ and 21 Controls	Microarray	let-7g, miR-181b		miR-181b	Visinin-like protein 1 (VSNL1, NM_003385) and Glutamate ionotropic receptor AMPA type subunit 2 (GRIA2, NM_000826) genes are potential target of miR-181b	(103)
**MDD**	ACC	15 MDD, 8 BD, and 14 Controls	qPCR		miR-34a, -184			(104)
	BA44	14 MDD and 11 Controls	qPCR		miR-1202		miR-1202 regulates the expression of Metabotropic glutamate receptor 4 (GRM4, NM_000841) and predicts antidepressant response	(105)
	BA46	15 MDD and 15 Controls	qPCR	miR-124-3p				(106)
**BD**	ACC	15 MDD, 8 BD, and 14 Controls	qPCR		miR-34a, -132 -133a, -212		There was inverse correlation between nuclear receptor coactivator 1 (NCOA1, NM_003743) and miRNA-34a	(104)
	ACC	5 BD and 6 Controls	qPCR	miR-149			Glial cell derived exosome samples	(107)
	BA9	8 SCZ, 9 BD, and 13 Controls	Microarray	miR-29c		miR-29c	Exosome samples	(95)
	BA46	35 SCZ, 35 BD, and 35 Controls	TLDA	miR-22, -133b, -145, -145*, -154*, -504, -889	miR-29a, -32, -140-3p, -454*, -520c-3p, -573, -767-5p, -874			(97)
	BA46	35 SCZ, 32 BD, and 34 Controls	qPCR	miR-346 was not significantly changed				(99)
	BA46	1^st^ set (7 SCZ, 9 BD, and 10 Controls) 2^nd^ set (35 SCZ, 34 BD, and 35 Controls)	qPCR	miR-137 was not significantly changed				(102)
	dlPFC	35 SCZ, 31 BD, and 34 Controls	Microarray	miR-32, -188-5p, -187, -196b, -297, -383, -490-5p, -449b, -513-5p, -876-3p				(101)
	Hippocampus dentate gyrus granule cells	15 SCZ, 15 MDD, 15 BD, and 15 Controls	RNA-seq	miR-182 was not significantly changed			Genotype of rs76481776 affects the miR-1842 expression	(108)
	Cerebellum	29 BD and 34 Controls	qPCR	miR-34a			Upregulation was also confirmed in induced neurons from human fibroblast	(109)

ACC, Anterior Cingulate Cortex; BA, Brodmann area; BD, Bipolar Disorder; dlPFC, Dorsolateral Prefrontal Cortex; miRNA, microRNA; MDD, Major Depressive Disorder; SCZ, Schizophrenia; STG, Superior Temporal Gyrus; TLDA, TaqMan Low Density Arrays.

### miRNAs and Schizophrenia

MiR-137 is a well-known miRNA in schizophrenia studies (110). The first report of the involvement of miR-137 came from a GWAS study which linked a risk variant rs1625579 within miR-137 to SCZ (111). A previous study had reported that target genes for miR-137 are associated with activation in the dlPFC (112). Guella et al. (102) subsequently examined miR-137 and its target genes in the dlPFC of SCZ and BD subjects. MiR-137 expression in the dlPFC was not different between diagnoses. Interestingly, when the genotype of rs1625579 was considered, significantly lower miR-137 expression was found along with higher expression of TCF4, a target gene of miR-137, in the homozygous TT subjects compared to TG and GG subjects within the control group. The miR-137 expression was region specific with amygdala and hippocampus having the highest levels. These results suggest that miR-137 and associated TCF4 gene may be risk factors for SCZ. Using miRNA-seq, one amygdala study showed seven upregulated and 11 downregulated miRNAs (73); of them, miR-1307 was located on a locus previously found to be associated with SCZ risk allele rs11191419 in GWAS (113).

Using custom microarray, Perkins et al. (93) studied miRNA expression in BA9 of subjects with SCZ or schizoaffective disorder and compared them with psychiatrically unaffected individuals. The study found 15 downregulated and one upregulated miRNA in these subjects. They further examined sequences in the pri-miRNA motifs of those dysregulated miRNAs and found that several miRNAs shared the same sequence. For example, miR26b, miR-30a, miR-30b, and miR-7-1 had UGAGNCUU upstream sequences, whereas miRNAs miR-9-1, miR-9-2, miR-9-3, miR-7-3, and miR-30e had GUCNCUUC upstream sequences in their corresponding pre-miRNAs. This suggests the possibility that the processing of pri-miRNA to pre-miRNA might be affected, which could eventually be related to miRNA expression changes. Moreau et al. (94) reported that 19% of miRNAs in BA9 were altered based on diagnostic classification among SCZ, BD, and control subjects. Interestingly, all of the downregulated miRNAs in SCZ were downregulated in BD subjects, supporting the similarity of the genetic background between SCZ and BD. Banigan et al. (95) explored miRNA expressions in exosomes of BA9. Based on the microarray data, they validated the upregulation of miR-497. Several studies have shown that exosomes in the CNS may play a role in neuronal communications, nerve regeneration, synaptic plasticity, and immune response (114, 115).

Beveridge et al. (96) detected 25 upregulated miRNAs in BA9 of SCZ subjects; of them, 10 miRNAs (let-7d, miR-128a, miR-16, miR-181b, miR-181a, miR-20a, miR-219, miR-27a, miR-29c, and miR-7) were validated with qPCR. They also revealed that the biogenesis of miR-181b was perturbed in SCZ possibly due to the altered expression of pri- and pre-miR-181b and miRNA biogenesis-related enzymes DGCR8, DROSHA, and DICER1. Kim et al. (97) detected 7 upregulated miRNAs in another prefrontal cortical area BA46 of SCZ subjects. Of them, miR-132 was highly expressed in the forebrain and was regulated by cAMP response element-binding (CREB) and extracellular signal regulated kinase (ERK) signaling. Both CREB and ERK are targets of antipsychotic drugs (91, 116). On the contrary, Miller et al. (101) reported downregulation of miR-132 in dlPFC of SCZ subjects. The authors found that NMDA antagonist, when administered to adult mice, resulted in lower expression of miR-132 in the prefrontal cortex. Since miR-132 is highly expressed in the postnatal period, a period of synaptic pruning thought to be related to the mechanism of neurodevelopmental susceptibility to SCZ through NMDAR signaling (91), it was concluded that dysregulation in miR-132 and associated target genes may contribute to the neurodevelopmental and morphological abnormalities shown in SCZ.

Santarelli et al. (98) investigated miRNA expression in BA 46 of subjects with SCZ and schizoaffective disorder. They found differential regulation of 28 miRNA in the SCZ group compared to controls. Of them, six miRNAs (miR-17, miR-107, miR-134, miR-328, miR-382, and miR-652) were validated by qPCR. They also found increased expressions of miRNA biogenesis enzymes DGCR8, DROSHA, and DICER1 and concluded that miRNA changes may occur due to disruption in mRNA biogenesis. In another study, Zhu et al. (99) found lower expression of miR-346 in BA46 of SCZ subjects. Interestingly, miR-346 is positioned in intron 2 of the glutamate receptor ionotropic delta 1 (GRID1) gene, which was found to be downregulated in SCZ subjects. GRID1 is an important gene related to SCZ susceptibility (117). Recently, temporal dynamics of miRNA expression in dlPFC as well and their dysregulation was studied in SCZ subjects (100). It was found that miRNAs that were enriched in infants were upregulated, and those enriched in prepuberty were downregulated in SCZ. The targets of these miRNAs included genes encoding for synaptic proteins and were found to be altered in SCZ subjects. In addition, a significant upregulation in the expression of miR-3162 and miR-936 was noted. These results suggest the miRNAs may participate in the development of dlPFC. In addition, miRNAs that are enriched in infancy and prepuberty may be critical in the deployment of SCZ pathology.

Two other reports which used superior temporal gyrus (STG) also linked miRNA abnormalities to SCZ (96, 103). Both studies showed upregulation of miR-181b in SCZ subjects. Using reporter assay, one study revealed that miR-181b could regulate VSNL1 and GRIA2 genes (103). Expression changes in GRIA2 have been reported in BA46 (118) and the medial temporal lobe (119, 120) of SCZ subjects.

### miRNAs and Major Depressive Disorder

A number of studies have linked miRNA changes to major depressive disorder (MDD) pathophysiology. Azevedo et al. (104) analyzed select 29 miRNAs by qPCR in ACC of subjects with MDD, BD, and control subjects. They found that miR-132, miR-133a, and miR-212 were differentially regulated in BD, miR-184 in MDD, and miR-34a in both MDD and BD. None of the miRNAs survived significance after multiple corrections. *In silico* analysis identified NCOA1, NCOA2, and PDE4B as a target of miR-34a, while NCOA2 and PDE4B were targeted by miR-184. Further qPCR analyses showed that NCOA1 had an inverse relationship with miR-34a in BD, while NCOR2 had a positive relationship with MDD. It is pertinent to mention that both NCOA1 and NCOR2 regulate the GR-mediated transcription of corticotropin-releasing hormone, a risk gene for stress-related psychiatric illnesses (121).

Lopez et al. (105) studied miRNA expression in the ventrolateral prefrontal cortex (vmPFC) of depressed individuals and found that primate-specific and human brain enriched miR-1202 was the most differentially regulated miRNA in MDD subjects. They found that miR-1202 regulated the expression of gene encoding metabotropic glutamate receptor-4 (GRM4). A recent report suggests that GRM4 3′ UTR variant (rs2229901) is associated with MDD risk (122). Interestingly, the antidepressant treatment induced miR-1202 expression and downregulated GRM4 expression (105). These results suggest that miR-1202 may be associated with MDD and may serve as target for new drug development.

It has been reported that miR-124-3p is highly expressed specifically in the brain (123), and has neuron-specific expression (124–126). Our group reported a significant upregulation in miR-124-3p expression in BA46 of depressed individuals (106). *In silico* prediction suggested that AMPA/NMDA receptors are major targets of miR-124-3p. Of them, expression changes for GRIA2 in dlPFC (127), GRIA3 in dentate gyrus (128) and PFC (129), and GRIA4 in PFC (129, 130) have been found in depressed individuals.

### miRNAs and Bipolar Disorder

Compared to MDD, *postmortem* brain studies of miRNAs in bipolar disorder are limited. Azevedo et al. (104) reported downregulation of 4 miRNAs (miR-34a, miR-132, miR-133a, and miR-212) in ACC of BD subjects. miR-34a was correlated with PDE4B expression in BD. PDE4B expression changes have been found in several psychiatric disorders including SCZ (131, 132), MDD (133), and BD (134). Another ACC (BA24) study conducted by Choi et al. (107) explored expression changes in five miRNAs (miR-29c, miR-31, miR-15b, miR-497, miR-219, and miR-149) in glial exosomes. They found that miR-149 was significantly upregulated in BD subjects. Since miR-149 inhibits glial proliferation, an increase in miR-149 may be associated with previously reported reduced glial cell numbers in BA24 of patients diagnosed with BD (135).

Banigan et al. (95) explored exosomal miRNA expression in BA9 of patients diagnosed with BD and found that miR-29c expression was significantly increased. miR-29c regulates canonical Wnt signaling (136). Wnt signaling is linked to BD pathogenesis such that lithium impairs GSK-3*β* expression, a component of Wnt signaling system (137). Kim et al. (97) investigated the expression of 667 miRNAs in the PFC of individuals with SCZ and BD using Taqman Low Density Array system. They found downregulation of seven miRNAs in SCZ and 15 miRNAs in BD. These miRNAs targeted genes within the networks overrepresented for neurodevelopment, behavior, and SCZ and BD development (97).

Genetic similarities between SCZ and BD have been well documented (138). The overlapping miRNA changes between SCZ and BD may explain the genetic similarity. In the study by Kim et al. (97), miR-154* showed downregulation in PFC of both SCZ and BD subjects. Kohen et al. (108) investigated transcriptomic changes in the dentate gyrus (DG) granule cells of hippocampus in subjects with SCZ, MDD, BD, and nonpsychiatric controls. One miRNA that stood out was miR-182. MiR-182 is involved in several biological processes such as immune response (139), DNA repair (140), and the regeneration of peripheral nerves after injury (141, 142), which play key roles in BD pathogenesis. Carriers of different genotypes among subjects with SCZ and MDD had a loss of DG miR-182 signaling. Interestingly, carriers of high (C/C) *vs* low-expressing genotype (C/T or T/T) of rs76481776 in both controls and BD subjects, which is located in miR-182, had different levels of miR-182 target gene expression. This suggests that miR-182 may play a critical role in shaping the DG transcriptome based on genotype. Bavamian et al. (109) studied miRNA expression changes in cerebellum and reported an increased expression of miR-34a in BD subjects. In addition, they showed an upregulation of miR-34a in induced neurons derived from BD human fibroblast collected from another cohort. Subsequently, they validated the BD risk genes ankyrin-3 (ANK3) and voltage-dependent L-type calcium channel subunit (CACNB3) as direct targets of miR-34a with luciferase reporter assay. Functionally, enhanced miR-34a expression reduced neuronal differentiation and altered neuronal morphology as well as expression of synaptic proteins. On the other hand, reduced miR-34a expression improved dendritic elaboration.

The studies mentioned above suggest that BD patients have a distinct miRNA expression profile; however, there is a significant overlap with SCZ. This is quite relevant. In the future, miRNA profiling may be useful in identifying commonality and distinction between these disorders, both functionally and at pathophysiological levels.

### miRNAs and Suicide

MiRNA studies related to suicide are summarized in **Table 4**. Using Taqman Low Density Array system, our group was the first to examine miRNA expression changes in BA9 of 18 MDD suicides and 17 nonpsychiatric control subjects (143). We found a global downregulation of miRNAs in MDD subjects. Altogether, we found significant downregulation in 21 miRNAs. Additional 29 miRNAs were also found to be downregulated by >30%; however, they did not reach statistical significance. When the regulation of these downregulated miRNAs was studied in detail, it was observed that many of them were encoded at the same or nearby chromosomal loci. In addition, they shared 5′-seed sequences. This suggests that miRNAs that were downregulated in MDD subjects had overlapping mRNA targets. Several instances were noted where miRNAs were found to be targeting the same genes. Some of the validated target genes included transcription factors, transmembrane and signaling proteins, growth factors, epigenetic modifiers, and apoptotic regulatory proteins. Predicted targets included ubiquitin ligases, CAMK2G, splicing factor NOVA1, and the GABA-A receptor subunit GABRA4. DNMT3b was another protein that was found to be targeted and further validated. DNMT3b is a key gene in epigenetic modifications and has been shown to be involved in MDD pathophysiology (152).

**Table 4:** miRNA expression changes in *postmortem* brain and blood samples of suicide subjects.

Brain areas	Sample size	Methods	miRNA expression changes		Successfully validated miRNAs by qPCR	miRNA roles and other findings	Citations
			Upregulation	Downregulation			
BA9	18 MDD suicide and 17 Controls	TLDA		miR-142-5p, -137, -489, -148b, -101, -324-5p, -301a, -146a, -335, -494, -20b, -376a*, -190, -155, -660, -130a, -27a, -497, -10a, -20a, -142-3p		DNA (cytosine-5-)-methyltransferase 3 beta (DNMT3b) protein expression was significantly correlated with miR-148b expression	(143)
BA9	10 MDD suicide and 9 Controls	qPCR	miR-30a, -30e	miR-200a			(144)
BA10	15 SCZ (4 suicide), 15 MDD (7 suicide), 15 BD (9 suicide), and 15 Controls	TLDA	miR-376a, -625	miR-152, -181a, -330-3p, -34a, -133b	miR-17-5p, -145-5p, -219-2-3p	Synaptosome samples	(145)
BA10	38 suicide (4 SCZ, 23 MDD, 1 BD, and 6 subjects who did not have psychopathology) and 17 Controls	qPCR	miR-185*, 491-3p			TrkB-T1 (TrkB isoform) is a potential target of miR-185* validated by *in vitro* experiments	(146)
BA44	15 MDD suicide and 16 Controls	qPCR	miR-139-5p, -195, -320c, -34c-5p			There were significantly correlations (miR-34c-5p and miR-320c *vs* Diamine acetyltransferase 1 [SAT1, NM_002970]; miR-139-5p and miR-320c *vs* Spermine oxidase [SMOX, NM_001270691])	(105)
BA44	1^st^ set (24 MDD suicide and 35 Controls) 2^nd^ set (11 MDD suicide and 12 Controls)	qPCR		miR-218		Deleted in Colorectal Carcinoma (DCC, NM_005215) is a potential target of miR-218	(147)
BA46	12 MDD non-suicide, 14 MDD suicide, and 12 Controls	qPCR	miR-19a-3p			The upregulation of miR-19a-3p was also found in PBMC	(29)
LC	9 MDD suicide and 10 Controls	TLDA	miR-17-5p, -20b-5p, -106a-5p, -330-3p, -541-3p, -582-5p, -890, -99-3p, -550-5p, -1179	let-7g-3p, miR-409-5p, -1197			(148)
Edinger-Westphal nucleus	16 MDD suicide and 21 Controls	qPCR	miR-511			GDNF family receptor alpha-1 (GFRα1, NM_001145453) is a potential target of miR-511 validated by *in vitro* experiment	(149)
vPFC	32 MDD suicide and 20 Controls	qPCR	miR-425-3p, -146-5p, -24-3p, -425-3p			Four miRNAs are associated with MAPK/Wnt signaling pathways	(150)
Leukocytes	10 MDD with suicidal ideation, 22 MDD without ideation, and 32 Controls	qPCR	miR-34b-5p and -369-3p				(151)

ACC, Anterior Cingulate Cortex; BA, Brodmann area; BD, Bipolar Disorder; LC, Locus Coeruleus; miRNA, microRNA; MDD, Major Depressive Disorder; PBMC, Peripheral Blood Mononuclear Cells; SCZ, Schizophrenia; TLDA, TaqMan Low Density Arrays; vPFC, Ventrolateral Prefrontal Cortex.

Gorinski et al. (144) examined whether 5-HT1A receptor (5HT1AR) palmitoylation is a mechanism involved in MDD and suicidality. They found that miR-30e expression was increased, while ZDHHC21 expression, which they identified as a major palmitoyl acyltransferase involved in palmitoylation of 5-HT1AR, was reduced in PFC of MDD suicide subjects, suggesting that 5-HT1AR palmitoylation may be key to MDD pathogenesis and treatment. Another mechanistic study by Torres-Berrio et al. (147) examined whether the expression of Netrin-1 guidance cue receptor DCC (deleted in colorectal cancer) gene was associated with resiliency or susceptibility to PFC dysfunctions in MDD suicide *via* miRNAs. They found a significant correlation between the decreased expression of miR-218 in BA44 of MDD suicide subjects and the elevated expression of DCC. By conducting an animal study, they concluded that miR-218 was associated with susceptibility *versus* resiliency to stress-related disorders through regulation of DCC expression.

Our group assessed miRNA expression in the synaptosome of BA10 from a cohort of SCZ, MDD, and BD subjects (145). Altogether, eight out of 14 BD subjects, seven out of 15 MDD subjects, and three out of 14 SCZ subjects had died by suicide. The result of comparison between all suicides (across diagnostic categories) and all non-suicide groups showed two upregulated miRNAs (miR-376a and miR-625) and six downregulated miRNAs (miR-152, miR-181a, miR-330-3p, miR-34a, and miR-133b). Based on significance values, miR-152 was found to be strongly associated with suicide. Distinct set of miRNAs were found in all three disorders; however, several miRNAs showed overlapping changes in SCZ and BD subjects. Discrete changes were also noted in MDD and suicide groups. Interestingly, downregulated miRNAs in SCZ group were enriched within the synapse. Follow-up studies in purified synaptosomes by deep sequencing in SCZ group suggested that there was a significant loss of small RNA expression in synaptosomes only for certain sequence lengths within the miRNA range. In addition, a large number of miRNAs (n = 73) were significantly downregulated and only one miRNA was upregulated. Remarkably, greater fold change of miRNAs in SCZ was associated with lower synaptic enrichment ratio in control subjects and vice-versa. Overall, synaptic miRNAs were generally downregulated in SCZ; however, highly synaptically enriched miRNAs showed greater down-regulation. It was concluded that there may be a deficit in synthesis, transport, or processing of miRNAs in SCZ; however, this process may be more selective for those miRNAs that are localized predominantly in the synaptic compartment.

Maussion et al. (146) examined miRNA expression changes in BA10 from 38 suicide subjects across SCZ, MDD, BD, and control subjects. miR-185* and miR-491-3p were found to be upregulated in suicide subjects compared to control subjects. TrkB-T1, one of the transcript variants of TrkB, expression was significantly lowered in suicide subjects and correlated with miR-185* expression. This was specific only for TrkB-T1, but not for TrkB-T2 or TrkB-FL. TrkB has been shown to be involved in suicidal behavior as has been reported by us previously (67). In an effort to reveal the regulation of polyamine system and suicidal behavior, Lopez et al. (153) examined the expression of SAT1 and SMOX in BA44 and found them to be lower in MDD suicide subjects. They selected 10 miRNAs which were predicted to regulate SAT1 and SMOX genes. Of those, four miRNAs (miR-34c-5p, miR-320c, miR-139-5p, and miR-320c) were significantly upregulated and correlated with the polyamine stress response genes (miR-34c-5p and miR-320c with SAT1; miR-139-5p and miR-320c with SMOX). They concluded that polyamine stress response genes including SAT1 and SMOX may be linked to alterations in miRNA networks that are responsive to stress in MDD suicide subjects. In another study, Lopez et al. (150) noted upregulation of miR-425-3p, miR-146-5p, miR-24-3p, and miR-425-3p in ventrolateral prefrontal cortex (vPFC) of MDD suicide subjects. Resulting from *in silico* prediction and *in vitro* experiments, they concluded that all four miRNAs were related to the MAPK/Wnt signaling pathways. These signaling systems have been implicated in suicidal behavior (154).

In an effort to examine the regulation of pro-inflammatory cytokine genes in suicidal behavior, we examined the relationship between TNF-α and its regulatory miRNAs (29). We found an increased expression of miR-19a-3p in BA46 of MDD individuals who had died by suicide. The increased expression of miR-19a-3p was also found in PBMC of MDD subjects who had suicidal ideation. Mechanistically, we found that RNA-binding protein HuR helped in stabilizing TNF-α transcript. This occurred apparently by sequestering its 3′ untranslated region from miR-19a-3p-mediated inhibition. The study suggests that miR-19a-3p may be involved in cytokine dysregulation in suicidal individuals. Recently, we also explored miRNA changes in the locus coeruleus (LC) of MDD suicide subjects (148). A total of 10 upregulated and three downregulated miRNAs were detected in these subjects. Based on target gene prediction using the upregulated miRNAs, we narrowed our study to those genes with a strong neuropsychiatric background. We focused on RELN, GSK-3*β*, MAOA, CHRM1, PLCB1, and GRIK1 and found reduced expression levels for RELN, GSK-3*β*, and MAOA.

Maheu et al. (149) examined glial cell line-derived neurotrophic factor in MDD suicide subjects and found an isoform-specific decrease in GDNF family receptor alpha 1 (GFRA1) mRNA, which was associated with lower GFR*α*1a protein levels in basolateral amygdala. They found upregulation in several miRNAs that may target GFRA1 gene. One of them was miR-511. Under the condition of miR-511 overexpression in neural progenitor cells (NPC), they observed that protein expression of GFR*α*1 was significantly decreased and concluded that GFR*α*1 is regulated by miR-511 and may be associated with suicidality.

In terms of blood studies associated with suicidality, only one study is available. Sun et al. (151) reported downregulation of miR-34b-5p and miR-369-3p in MDD subjects with suicidal ideation compared to MDD without suicidal ideation. These changes were consistent with our previous *postmortem* study conducted in BA9 of MDD suicide subjects (143).

## Piwi-Interacting RNAs

Piwi-interacting RNAs (piRNA) are the large small non-coding RNAs (26–32 nucleotide), which are preferentially expressed in nuclei and cytoplasm (155). Piwi proteins are recognized as one domain of argonaute (Ago) protein and bind to piRNA (156–159). The functions of Piwi/piRNA are unclear; however, they are highly expressed in germlines such as testes (156) and ovaries (160). Several studies show their role in epigenetic regulation of transposable elements in germlines (161–163). The existence of piRNA in mouse hippocampal neuron was reported by Lee et al. (164). In an *Aplysia* study, Rajasethupathy et al. (24) found that neuronal piRNA has a role in regulating memory-related synaptic plasticity. They found that the Piwi/piRNA complex may enable serotonin-dependent methylation of a CREB2 promoter CpG island in neurons, leading to the enhancement of long-term synaptic facilitation, learning-related synaptic plasticity and memory storage in *Aplysia*. More recently, Nandi et al. (165) reported that piRNA of Mili, the mouse ortholog of Piwi, exhibited behavioral deficits such as hyperactivity and less anxiety (165).

There is limited direct evidence of piRNA in psychiatric disorders. One study identified 37 piRNAs in ACC of SCZ subjects and one piRNA was correlated with antipsychotic medication (166). However, the authors did not mention the expression change between SCZ and control subjects because most of piRNAs were expressed only in a small number of samples.

## Small Interfering RNAs

Small interfering RNAs (siRNA) are short (~20-24 nucleotide) double-stranded RNAs (dsRNAs) and interfere in the translation of proteins. Dicer enzyme cleaves long dsRNA and small hairpin RNAs into siRNAs (167, 168). SiRNAs have a role in mRNA cleavage by guiding RNA-induced silencing complex (RISC) to its complementary target mRNAs. Initially, siRNAs are loaded into Ago2 as duplexes. Subsequently, passenger strand of siRNAs is cleaved by Ago2; then, guide strand from siRNA duplex is liberated and produces active RISC, which has the capability to cleave target mRNAs (169, 170). Mainly, siRNAs are used for suppressing target genes as a scientific experimental method. To our knowledge, there is no report about siRNA expression changes in psychiatric disorders.

## Small Nuclear RNAs

Small nuclear RNAs (snRNA), averaging about 150 nucleotides in length, are one of the components of spliceosomes. Five snRNAs (U1, U2, U4, U5, and U6) have been identified. snRNAs form ribonucleoprotein complexes (snRNPs) that can target and bind to specific sequences on pre-mRNAs (171). Spliceosomes have key roles in nuclear pre-mRNA splicing that remove the pre-mRNA regions (intron) that do not code for functional molecules. Initially, U1 snRNA binds to its associated proteins at the 5′ splice end of the hnRNA (172). Subsequently, U2 snRNP is recruited to the spliceosome binding site for making complex A. Bai et al. (173) reported that unique U1 snRNP pathology is involved in abnormal RNA splicing in Alzheimer’s disease (AD) that leads to the alteration in amyloid precursor protein (APP) expression and A*β* levels. Their group also showed U1 snRNP pathologic changes in *postmortem* brain of early onset AD (174). In addition, the small nuclear ribonucleoprotein U1-70K is aggregated in AD brain (175) and might contribute to neuronal toxicity in conjunction with N-terminal truncation (N40K) fragment derived from cleavage (176). In a mouse study, it is reported that dysfunction of U2 snRNA causes neurodegeneration through distortion of pre-mRNA splicing (177). A positron emission tomography study showed that amyloid accumulation is found in the brain of late-life MDD patients (178). At this stage, amyloid deposition may occur during the preclinical phase of dementia in patients who have depressive symptoms (179, 180), although MDD itself is a risk factor for the onset of AD (181). It is possible that A*β* changes through snRNAs may occur in late-life MDD patients even though the relationship between MDD and A*β* is unclear. One study, using ACC, found that 149 snRNAs were expressed in both SCZ and control subjects, and 35 snRNAs were expressed specifically for SCZ subjects (166). However, their biological impact on SCZ pathogenesis is unclear.

## Small Nucleolar RNAs

Small nucleolar RNAs (snoRNAs) are generally responsible for guiding modifications in rRNAs, tRNAs, and snRNAs. snoRNAs are divided into two main classes: 1) box C/D and 2) box H/ACA. Box C/D snoRNAs, averaging 60–90 nucleotides, have a role in catalyzing 2′-O-ribose methylation. Box H/ACA snoRNA, averaging 120–140 nucleotides, guide pseudouridylation (182, 183). In 2002, brain specific C/D box snoRNAs HBII-52 (SNORD115) and HBII 85 (SNORD116) and H/ACA box snoRNA HBI-36 were detected (184). Prader–Willi syndrome (PWS) is a neurodevelopmental disorder caused by imprinted gene clusters at human chromosome region 15q11q13. It was reported that SNORD115 and SNORD116 are partially responsible for the development of PWS (185, 186). Moreover, HBI-52 and HBI-36 snoRNAs are thought to regulate 5-HT_2c_ mRNA expression (184). 5-HT_2c_ has a role in MDD pathogenesis. Indeed, several antidepressants work as an antagonist to 5-HT_2c_ (187–189). In terms of schizophrenia, it is considered that 5HT-_2c_ relates to SCZ pathogenesis through the dopaminergic pathway (190, 191) and by causing side effects to antipsychotic treatment (192). Actually, clozapine, a drug used for refractory schizophrenia, has high affinity to 5-HT_2c_ (192). SnoRNAs may influence psychiatric disorders directly or through 5-HT_2c_ receptors. Epigenetic changes have also been found in SNORD115 and SNORD116 in monozygotic twin study of SCZ. Hypermethylation in 12 regions on SNORD115 and five regions on SNORD116 regions were found in affected individuals (193).

In PFC synaptosomes from SCZ subjects, we noted ~50% decrease in a set of sequences that were derived from a C/D box snoRNA and SNORD85 (145). A 27-mer sequence TTCACTGATGAGAGCATTGTTCTGAGC was the most abundant one that incorporated a C/D box and terminated four bases before the 3′ end of the host snoRNA. Our study, for the first time, showed that not only is this novel class of small ncRNAs expressed in humans, and more so in synaptosomes, but they may be significantly altered in a psychiatric disorders. Another study also showed expression change in snoRNAs in SCZ (166). A total of 343 snoRNAs were expressed in both SCZ and control subjects and six snoRNAs were expressed specifically in SCZ subjects. However, none of the snoRNAs were significantly changed in SCZ compared to controls.

## Limitations

There are a several limitations to ncRNA studies in neuropsychiatric disorders: 1) Several studies show changes in expression of expression lncRNAs and miRNAs; however, they are inconsistent even when studied in the same brain region with the same diagnosis. This could be attributed to several factors. For example, confounding variables such as PMI, brain pH, freeze–thaw cycle, age, and sex vary between studies. In addition, most of the studies use whole tissue. As mentioned earlier, the expression of lncRNAs and miRNAs are brain region and cell type specific. This cellular heterogeneity may affect ncRNA expressions. Thus, studying their expression patterns in neurons and glial cells (oligodendrocytes, astrocytes, and microglia) may delineate these discrepancies. Even within neurons, specific neuronal population needs to be studied. In this regard, single cell study will be helpful. 2) In recent years, many studies have focused on miRNAs and to a certain extent lncRNAs; however, the role of siRNAs, piRNAs, snoRNAs, and snRNAs are understudied. Given their important roles (*e.g.* epigenetic regulation of transposable elements; mRNA cleavage by guiding RISC), future research will be needed to explore their functional significance in psychiatric disorders. 3) Correct identification of new snRNAs and snoRNAs and their epigenetic roles needs to be studied (194, 195). New technologies like nanopore sequencing and structure prediction algorithms are emerging that can be helpful in this regard. 4) Although new members of ncRNA are being discovered regularly, the functions of these novel ncRNAs are not well understood. More *in-vivo* functional studies followed by high-throughput sequencing after chemical cross-linking or photoactivatable cross-linking and immunoprecipitation (PAR-CLIP) will be effective in probing the functional roles of novel ncRNAs at the cellular level (196). These assays can be also performed in human *postmortem* brain samples, but by minimizing the loss of tissue integrity and maximizing the likeliness of capturing the interaction at the molecular level. 5) Most of the studies have shown the temporal changes in ncRNA expression. Although it is very difficult to measure ncRNA expressions at different time points in *postmortem* samples, it is relatively easy to measure them in peripheral tissue samples. Using longitudinal studies, it will be possible to explore the diagnosis/therapeutic/disease state biomarkers for psychiatric illnesses.

## Conclusions

**Figure 3:**
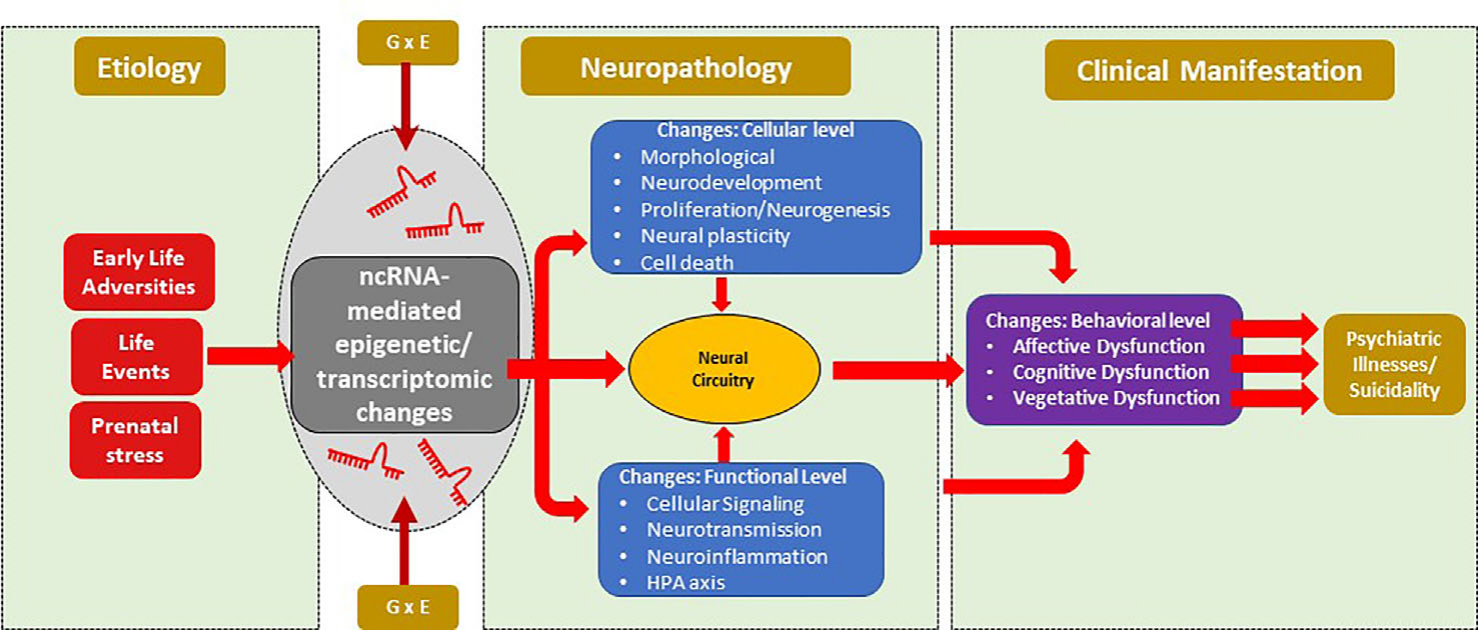
Schematic diagram of the non-coding RNAs’ impact on psychiatric illnesses and suicidality. Risk factors for mental illnesses include early life adversity, current or recurrent life events that along with gene environment interaction can lead to epigenetic modifications mediated by ncRNAs. These modifications can give rise to neuropathology mediated by changes at cellular and/or molecular levels, which can subsequently alter neural circuitry. Phenotypic changes can arise from circuitry changes that can mediate the development of psychiatric illnesses such as major depression, bipolar disorder and schizophrenia. Suicidal behavior could be a manifestation of psychiatric illnesses or may be independent of psychiatric illnesses. ncRNAs, non-coding RNA.

ncRNAs are gaining traction for their role in various psychiatric illnesses. A schematic diagram of the non-coding RNAs’ impact on psychiatric illnesses and suicidality is depicted in **Figure 3**. Both long-non-coding RNAs and miRNAs have been extensively studied in MDD, SCZ, and BD. As can be seen in **Tables 1** **–** **4**, a large number of miRNAs and lncRNAs have been implicated in SCZ, MDD, and BD. There were several miRNAs that showed overlapping changes between SCZ and BD, suggesting that these two disorders may share common susceptibility factors as has been demonstrated in several genetic studies.

The population-based studies have explored the expression changes of lncRNA and miRNA among psychiatric patients who have suicidal ideation. A couple of *postmortem* brain studies have also explored suicide associated ncRNAs. Interestingly, both lncRNAs and miRNAs were specifically changed in psychiatric patients who died by suicide even though the sample size and number of studies are quite small. The results are encouraging and need further exploration.

In terms of other small RNAs, the studies are quite limited; nevertheless, they appear to be promising. piRNAs and snoRNAs show interesting results in SCZ patients that may shed light on their role in regulating other RNAs and their role in this psychiatric illness. These studies need to be expanded in order to understand their precise role in regulatory mechanisms that can be involved in the pathogenesis of psychiatric disorders.

## Author Contributions

YD conceptualized, outlined, and edited the paper. YY reviewed literature and co-wrote the paper.

## Funding

This work was supported by funding from the National Institutes of Health (R01MH082802; R01MH101890; R01MH100616; R01MH107183; R01MH118884) to YD.

## Conflict of Interest

The authors declare that the research was conducted in the absence of any commercial or financial relationships that could be construed as a potential conflict of interest.

## Glossary

**Table:**

ncRNA	Non-coding RNA
lncRNA	Long non-coding RNA
Nt	Nucleotide
miRNA	microRNA
siRNA	Small interfering RNA
piRNA	Piwi-interacting RNA
snoRNA	Small nucleolar RNA
snRNA	Small nuclear RNA
SCZ	Schizophrenia
MDD	Major depressive disorder
BD	Bipolar disorder
bp	Base-pair
Xist	X-inactive specific transcripts
cDNA	Complementary DNA
Brn 1b	Brain-specific homeobox/POU domain protein 1b transcript
RMST	Rhabdomyosarcoma 2 associated transcript
TUNA	Tcl1 upstream neuron-associated transcript
Evf2	Erwinia virulence factor 2 transcript
GABA	Gamma aminobutyric acid
Pnky	Pinky
Bdnf	Brain-derived neurotrophic factor
BDNF-AS	Brain-derived neurotrophic factor antisense RNA
GWAS	Genome-wide association study
CNNM2	Cyclin and CBS domain divalent metal cation transport mediator 2
ACC	Anterior cingulate cortex
dlPFC	Dorsolateral prefrontal cortex
RNA-seq	RNA sequencing
IRF2	Interferon regulatory factor 2
LY6E	Lymphocyte antigen 6E precursor
HMOBX1	Homeobox containing 1
REEP5	Receptor accessory protein 5
PTPRT	Protein tyrosine phosphatase receptor type T
NFATC3	Nuclear factor of activated T cells 3
CNS	Central nervous system
MARCKS	Myristoylated alanine-rich C-kinase substrate
eQTL	Expression quantitative trait locus
WGCNA	Weighted correlation network analysis
P2RY13	Purinergic receptor P2Y13
PBMC	Peripheral blood mononuclear cell
qPCR	Quantitative polymerase chain reaction
p-GSK3β	Phosphorylated glycogen synthase kinase 3 beta
SERT	Serotonin transporter
pri-miRNA	primary microRNA
pre-miRNA	precursor microRNA
DGCR8	DiGeorge syndrome critical region 8
XPO5	Exportin-5
TRBP	TAR RNA-binding protein
Ago	Argonaute
RISC	RNA-induced silencing complex
p250GAP	p250 GTPase-activating protein
MeCP2	methyl CpG binding protein 2
TCF4	Transcription factor 4
BA	Brodmann area
CREB	cAMP response element-binding
ERK	Extracellular signal regulated kinase
NMDA	N-methyl-D-aspartate receptor
GRID1	Glutamate receptor ionotropic delta 1
STG	Superior temporal gyrus
VSNL1	Visinin like 1
GRIA2	Glutamate ionotropic receptor AMPA type subunit 2
GRIA3	Glutamate ionotropic receptor AMPA type subunit 3
GRIA4	Glutamate ionotropic receptor AMPA type subunit 4
NCOA1	Nuclear receptor coactivator 1
NCOA2	Nuclear receptor coactivator 2
PDE4B	Phosphodiesterase 4B
NCOR2	Nuclear receptor corepressor 2
GR	Glucocorticoid receptor
vmPFC	Ventromedial prefrontal cortex
GRM4	Glutamate receptor-4
AMPA	*α*-amino-3-hydroxy-5-methyl-4-isoxazolepropionic acid receptor
DG	Dentate gyrus
ANK3	Ankyrin-3
CACNB3	Calcium voltage-gated channel auxiliary subunit beta 3
CAMK2G	Calcium/Calmodulin dependent protein kinase II gamma
NOVA1	Neuro-oncological ventral antigen 1
GABA-A	Gamma-aminobutyric acid type A receptor subunit alpha
GABRA4	Gamma-aminobutyric acid type A receptor subunit alpha 4
DNMT3b	DNA (cytosine-5-)-methyltransferase 3 beta
5HT1AR	5-Hydroxytryptamine receptor 1A
ZDHHC21	Zinc Finger DHHC-type palmitoyltransferase 21
DCC	Deleted in colorectal carcinoma netrin 1 Receptor
TrkB	Tropomyosin receptor kinase B
TrkB-T1	Tropomyosin receptor kinase B transcript isoform 1
TrkB-T2	Tropomyosin receptor kinase B transcript isoform 2
TrkB-FL	Tropomyosin receptor kinase B full-length isoform
SAT1	Spermidine/Spermine N1-acetyltransferase 1
SMOX	Spermine oxidase
vPFC	Ventrolateral prefrontal cortex
MAPK	Mitogen-activated protein kinase
Wnt	Wingless and Int-1
TNF-α	Tumor necrosis factor alpha
HuR	Human antigen R
LC	Locus coeruleus
RELN	Reelin
MAOA	Monoamine oxidase A
CHRM1	Cholinergic receptor muscarinic 1
PLCB1	Phospholipase C beta 1
GRIK1	Glutamate ionotropic receptor kainate type subunit 1
GFRA1	GDNF family receptor alpha 1
CREB2	Cyclic AMP response element-binding protein 2
Mili	Miwi like; Piwil2
dsRNAs	Double-stranded RNAs
Ago2	Argonaute 2
snRNPs	Small nuclear ribonucleoprotein complexes
AD	Alzheimer’s disease
APP	Amyloid precursor protein
Aβ	Amyloid beta
N40K	N-terminal truncation fragment
HBII-52	C/D box snoRNA 52
HBII-85	C/D box snoRNA 85
HBI-36	H/ACA box snoRNA 36
PWS	Prader–Willi syndrome
5-HT2c	5-Hydroxytryptamine receptor 2C
PMI	*Postmortem* interval
PAR-CLIP	Photoactivatable cross-linking and immunoprecipitation
